# Clinical impact of MRI-based risk calculators for prostate cancer diagnosis: a systematic review and meta-analysis

**DOI:** 10.1038/s41391-025-01014-2

**Published:** 2025-08-26

**Authors:** Ciarán Courtney O’Toole, Nancy Fosua Boakye, Ailish Hannigan, Amirhossein Jalali

**Affiliations:** 1 School of Medicine, University of Limerick, Limerick, Ireland; 2 Health Research Institute, University of Limerick, Limerick, Ireland; 3 Limerick Digital Cancer Research Centre, University of Limerick, Limerick, Ireland; 4 Research Ireland Centre for Research Training in Foundations of Data Science, Department of Mathematics and Statistics, University of Limerick, Limerick, Ireland

**Keywords:** Prostate cancer, Translational research

## Abstract

**Background:**

Prostate cancer (PCa) is the second most common cancer among men worldwide. Current diagnostic methods often lack sufficient sensitivity and specificity, leading to unnecessary biopsy. With growing use of MRI and EAU guideline recommendations, this review synthesised evidence on MRI-based risk calculators (RCs) for PCa diagnosis and compared their performance with traditional clinical RCs.

**Methods:**

A systematic search of Embase, Medline, Scopus, Cochrane Library, and Web of Science databases assessed the discriminatory ability of MRI-based RCs using Area Under the Curve (AUC). A meta-analysis was conducted to pool AUC estimates, assess heterogeneity, and compare the differences in discriminatory ability.

**Results:**

Of 2049 papers, 16 met the inclusion criteria. MRI-based RCs showed increased discrimination, with an AUC of 0.84 (95% CI: 0.81–0.86) for clinically significant PCa (csPCa), compared to 0.76 (95% CI: 0.73–0.79) for clinical models, and an AUC of 0.81 (95% CI: 0.78–0.84) for all PCa, compared to 0.74 (95% CI: 0.68–0.79). The pooled logit(AUC) difference was 0.49 units for csPCa and 0.37 units for all PCa. High heterogeneity was noted, likely due to PCa variability, and 31% of the studies had a high or unclear risk of bias, potentially affecting generali**s**ability.

**Conclusions:**

MRI-based RCs improve the diagnostic accuracy for PCa with the potential to reduce unnecessary biopsies and optimi**s**e healthcare resources, thereby supporting their integration into clinical practice.

## Introduction

In 2020, over 1.4 million new prostate cancer (PCa) cases and 375,000 deaths were reported globally [1, 2], making it the most common non-cutaneous cancer in men. Early and accurate diagnosis is essential to improve patient outcomes and reduce overtreatment, yet current diagnostic pathways are not without limitations.

Traditional diagnostic approaches, including prostate-specific antigen (PSA) testing, digital rectal examination (DRE), and biopsy often lack sensitivity and specificity, leading to overdiagnosis indolent cancers and unnecessary treatment [3–5]. PSA elevation is not cancer-specific and may result from benign conditions, necessitating further tests to avoid unnecessary intervention [6].

Currently, there is a high incidence of false-positive unnecessary prostate biopsies (approximately 15%) [7]. Leading to unnecessary stress for patients and their families and exposing them to risks of the biopsy procedures ranging from minor complications (e.g. haematuria, haematospermia) to serious infections, which account for 81% of biopsy-related hospitalisations [8]. These risks underscore the need for more selective diagnostic strategies.

To address these limitations, the European Association of Urology (EAU) has published new guidelines recommending advanced imaging techniques such as MRI and the use of multivariate risk stratification tools in the diagnosis of PCa [9]. Multiparametric MRI (mpMRI) has become a valuable clinical tool for PCa detection and evaluation enabling targeted biopsies (TBx) that significantly improve the detection of clinically significant cancers (csPCa–Gleason grade ≥ 3 + 4), reducing the diagnosis of indolent disease [10, 11].

Prostatic MRIs are graded using the Prostate Imaging Reporting and Data System (PI-RADS), which assesses csPCa likelihood on a five-point scale [12]. A PI-RADS score of one indicates that csPCa is highly unlikely, while a score of five suggests a high likelihood of significant disease [13]. Clinical decisions based on PI-RADS typically stratify patients into three risk categories: scores of 1–2 warrant surveillance, score 3 is equivocal and requires further evaluation, and scores of 4–5 usually prompt biopsy [14].

Beyond MRI, multivariable risk calculators (RCs) offer an additional layer of decision support. RCs are statistical models that estimate csPCa probability by integrating clinical and demographic variables. They support personalised decision-making, reduce unnecessary biopsies, and help standardise clinical practice [15]. Traditionally, clinical RCs have used variables such as age, PSA, family history, and DRE findings. Recently, MRI-based RCs have emerged, integrating mpMRI with traditional risk factors to improve diagnostic discrimination over MRI or clinical models alone [16, 17].

Despite these advances, a lack of high-quality evidence synthesising the performance of MRI-based RCs remains. A recent systematic review by Denijs et al. [18] provided a broad overview of existing RCs for PCa detection; however, no meta-analysis to date has comprehensively assessed the added value of mpMRI within RCs or directly compared the discriminatory performance of MRI-based and clinical calculators across studies.

To address this gap, we conducted a systematic review and meta-analysis comparing the discrimination performance (e.g. AUC) of MRI-based and clinical RCs within the same populations. We applied a novel meta-analytic approach to quantify the incremental value of mpMRI within multivariable prediction models and assess consistency across studies and settings. By synthesising the current evidence, our study aims to inform clinical decision-making and guideline development in PCa diagnostics.

## Methods

This systematic review and meta-analysis was conducted according to the Preferred Reporting Items for Systematic Reviews and Meta-Analyses (PRISMA) 2020 [19].

### Search strategy

Detailed search strategies were developed for five electronic repositories. The Embase, Medline, Scopus, and Cochrane Library databases and the Web of Science platform were searched to identify relevant literature for review. The protocol for this review was registered with PROSPERO (CRD42023475885) [20].

No date restrictions were applied; the search was limited to English. A search string was developed based on database exploration and key papers, using terms such as ‘PCa,’ ‘MRI,’ ‘AUC,’ ‘diagnosis,’ and ‘risk calculator.’ The full search strategy, including detailed search strings and Boolean operators, is available in the Supplementary File.

### Selection criteria and study selection

Studies were included if they compared the performance of an MRI-based risk calculator (or nomogram) with that of a non-MRI risk calculator involving men suspected of having PCa who underwent PSA screening as part of their diagnostic evaluation. Only peer-reviewed English-language papers, full conference proceedings, and studies presenting empirical data with full-text availability were considered.

Blinded screening was conducted using Rayyan screening software [21], by two independent reviewers (CCOT, NFB), with conflicts resolved by two additional reviewers (AJ, AH).

### Synthesis

Data extraction was conducted using Microsoft Excel following the Critical Appraisal and Data Extraction for Systematic Reviews of Prediction Modelling Studies (CHARMS) checklist [22]. All extraction was conducted by CCOT and reviewed by AJ, with conflicts resolved as needed.

### Statistical analysis

The analyses expanded on the original protocol [20], incorporating a meta-analysis to assess the added discrimination ability of MRI-based RCs over clinical RCs.

The effect size of interest in this study was the added discriminative ability of MRI in combination with clinical risk factors for PCa diagnosis, which was measured by the difference in AUC of the MRI-based RC over clinical RC. A meta-analysis was conducted on all the included studies with available data.

The pooled effect size was calculated using the AUC for MRI-based RCs and clinical RCs for both csPCa and all PCa using the Metamisc package in R [23]. Forest plots were created using the Valmeta function. Heterogeneity was assessed using *I*^2^ statistic and Cochran’s *Q* test.

To assess the added discrimination ability of MRI-based RC over clinical RC, the AUCs were logit-transformed [24] to enhance normality [25]. The difference in logit(AUC) was then obtained, and a random-effects model was fitted using the nlme package in R [26] to account for heterogeneity between cohorts. The model was weighted by the number of events in the validation sample, as this is a key determinant of study precision [27].

Publication bias was assessed using funnel plots and Egger’s test in meta-analyses with 10 or more studies.

Subgroup analyses were conducted to explore the influence of PI-RADS 3 proportion, sample size and validation type on the discriminatory ability of MRI-based RCs over clinical RCs for csPCa. Due to a limited number of studies presenting their results separately for these cohorts on biopsy naïve and prior biopsy patients was not conducted.

All analyses were conducted using R version 4.3.3 [28].

### Risk of bias assessment

Risk of bias was assessed using the Risk Of Bias ASsessment Tool (PROBAST) for prediction models [29] by two independent reviewers, with conflict resolution as needed.

## Results

### Search results

**Fig. 1:**
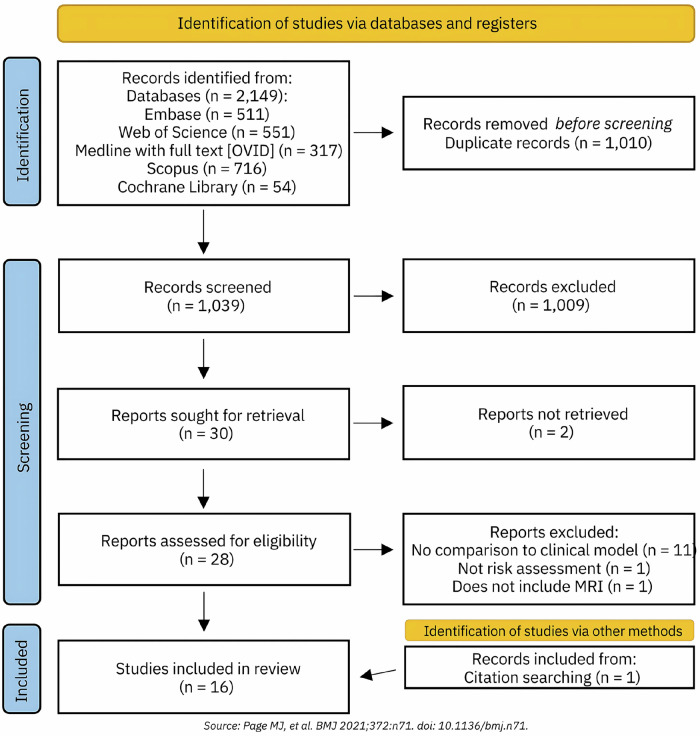
PRISMA Flow Diagram.

Searches yielded 2149 records of which 1010 duplicates were removed. 1039 records remained for screening. A total of 1009 records were excluded based on title and abstract screening. Full-text articles were sought for the remaining 30 reports, and two were not retrieved as they were conference abstracts. 28 reports were assessed for inclusion and 13 were excluded because they did not meet the inclusion criteria. The reasons for exclusion are shown in the PRISMA flow diagram (Fig. 1). An additional article was identified after searching for citations of the excluded review papers. A total of 16 studies [30–45] were included in the present review.

### Study characteristics

Study characteristics are summarised in Table 1. The studies were published between 2016 and 2023. Of the 16 studies, one was prospective and 15 were retrospective. Six studies were based in Europe, 6 in North America, 2 in Asia, and 2 in Australia. Sample sizes ranged from 221 to 2363 (median: 553; IQR: 363.8–974.5). Twelve studies developed a novel MRI-based RC model [30–33, 35–37, 39, 40, 42, 44, 45], four studies externally validated existing models [34, 38, 41, 43].

**Table 1:** Study characteristics.

Author, Year	Study design (cohort)	Study setting	Age, years	PSA level, ng/mL	PI-RADS 3-5	PI-RADS 3	All PCa	csPCa
					*n* (%)	*n* (%)	*n* (%)	*n* (%)
Alberts, 2018 [30]	Retrospective	Germany & Netherlands	66.0 (60.0–71.0)^a^	8.7 (6.1–12.9)^a^	298 (31)	123 (12.8)	491 (37.59)	345 (26.42)
Bhat, 2019 [31]	Retrospective	USA	64.7 (7.6)^b^	9.4 (13.0)^b^	303 (81.45)	77 (20.7)	Not reported	154 (41)
Falagario, 2021 [32]	Retrospective	Italy	66.0 (60.0, 71.0)^c^	5.5 (4.1, 7.4)^c^	59 (33.1)	35 (15.8)	119 (53.8%)	60 (27.1)
Fang, 2016 [33]	Retrospective	China	69 (62–75)^a^	10.7 (7.1–16.6)^a^	570 (63.8)	193 (21.6)	434 (48.5)	218 (24.2)
Hagens, 2022 [34]	Retrospective	Netherlands	67 (61–72)^a^	7.6 (5.3–11.3)^a^	1399 (88.8)	331 (21)	996 (63.2)	656 (41.7)
Kim, 2016 [35]	Retrospective	USA	63.9 (7.6)^b^	10.2 (15.1)^b^	199 (59.0)^d^	Not reported	Not reported	115 (34.0)
Kinnaird, 2022 [36]	Retrospective	USA	65 (7.8)^b^	6.5 (4.7, 9.5)^a^	1902 (81)	677 (29)	1408 (60)	942 (40)
Mehralivand, 2018 [37]	Retrospective	USA	64.3 (7.1)^b^	6.6 (4.7–9.5)^a^	367 (91.8)	53 (13.3)	272 (68.2)	193 (48.3)
Mortezavi, 2020 [38]	Retrospective	Sweden & Norway	64 (7)^b^	6.1 (4.1–8.8)^b^	429 (81)	103 (19)	291 (54.7)	194 (36.5)
Parekh, 2022 [39]	Retrospective	USA	65.0 (59.7, 70.4)^a^	5.2 (3.7, 7.7)^a^	1632 (69.1)	416 (17.6)	1370 (59)	742 (31.40)
Patel, 2023 [40]	Retrospective	USA	66 (61–70)^a^	6.3 (4.8–9.3)^a^	841 (84)	309 (31)	322 (47.8)	208 (30.9)
Radtke, 2017 [41]	Retrospective	Germany	65 (60–71)^a^	7.3 (5.4–10.6)^a^	934 (85)	367 (33)	732 (63)	489 (42)
Radtke, 2019 [42]	Retrospective	Germany & UK	65 (58–70)^a^	7.2 (5.0–11.5)^a^	259 (88)	127 (40)	176 (60)	114 (39)
Tay, 2021 [43]	Retrospective	Australia	65 (58.5–69)^a^	6.6 (4.7–9.5)^a^	123 (59.7)	41 (19.9)	163 (49)	96 (28.91)
van Leeuwen, 2017 [44]	Prospective	Australia	62 (55–67)^a^	5.5 (3.9–7.6)^a^	243 (61.8)	Not reported	227 (57.8)	149 (37.9)
Zhang, 2020 [45]	Retrospective	Taiwan	69.9 (8.4)^b^	11(7.1–19.1)^a^	320 (55.8)	98 (17.1)	408 (72)	165 (28)

^a^Median (IQR).

^b^Mean (sd).

^c^Median (min-max).

^d^PI-RARDS 4-5 only, 3 not reported.

The average age of the cohort ranged from 62 to 70 years old. The average PSA levels ranged from 5.2 to 11.0 ng/ml. The proportion of PI-RADS 3–5 ranged from 31 to 91.8%, with a median of 69.7%. The PI-RADS 3 proportion ranged from 12.8 to 40% with a median of 19.5%. Twelve studies reported the prevalence of PCa in their study population, with a mean prevalence of 57.6% (range 47.8–72.0%). All studies reported the prevalence of csPCa in their study population with a mean of 35.0% (range 24.4–48.3%).

### Study performance

All RCs were developed using logistic regression. Table 2 outlines the model performance measures and Table 3 the model predictors. Four studies divided their sample sizes for model application: three split their samples based on biopsy status and applied the models separately to groups with prior biopsies and those who were biopsy-naïve, and one study divided the samples by study site and treated the two sites as separate cohorts. The number of final predictors included in the MRI-based RCs ranged from four to eight, with a median of seven. Five (31%) studies performed internal validation of their models, three (19%) combined internal validation with external validation, and four (25%) performed only external validation. Calibration was assessed in 11(69%) studies: four (25%) used graphical plots only, and seven (44%) combined graphical and numerical methods. All studies assessed discrimination by AUC or C-statistic.

**Table 2:** Study Performance.

Author, Year	Novel Model	Sample size	Events	Missing Data *n* (%)	Cohort [model]	Models AUC for csPCa		Internal Validation Method	Performance measure(s)	Callibration
			*n* (%)			AUC_clin	AUC_MRI			
Alberts, 2018	Yes	504	213 (42.3)	441 (33)	Bx naïve	0.757	0.843	Bootstrapping	AUC & DCA	Yes
		457	132 (28.9)		Prior Bx	0.742	0.85			
Bhat, 2019	Yes	372	154 (41.4)	N/I	[Clin: PCPT]	0.7	0.76	N/I	AUC	No
					[MRI: PCPT + MRI]					
Falagario, 2021	Yes	221	60 (27.1)	N/I	[Clin: FPC-RC]	0.801	0.87	N/I	AUC & DCA	No
					[MRI: FPC-RC-MRI]					
Fang, 2016	Yes	894	218 (24.4)	N/I	All	0.85	0.872	N/I	c-statistic & DCA	Yes
Hagens, 2022	No (Ex Val)	1575	656 (41.7)	N/I	All	0.75	0.79	N/A	AUC & DCA	Yes
Kim, 2016	Yes	185	63 (35.1)	N/I	Bx naïve	0.6	0.72	N/I	AUC	No
		154	52 (33.8)		Prior Bx	0.61	0.63			
Kinnaird, 2022	Yes	2354	942 (40.0)	903 (38.4)	All	0.757^a^	0.888^a^	N/I	AUC	No
Mehralivand, 2018	Yes	400	193 (48.3)	0 (0.0)	All	0.640^a^	0.840^a^	N/I	AUC & DCA	Yes
Mortezavi, 2020	No (Ex Val)	532	193 (36.3)	N/I	[Clin: ERSPC]	0.8	0.87	Bootstrapping	AUC & DCA	Yes
					[MRI: Alberts]					
Parekh, 2022	Yes	2363	742 (31.4)	161 (6.8)	All	0.78	0.82	N/I	AUC & DCA	Yes
Patel, 2023	Yes	674	208 (30.9)	124 (18.4)	All	0.694^a^	0.839^a^	N/I	AUC & DCA	Yes
Radtke, 2017	Yes	670	318 (47.5)	15 (1.3)	Bx naïve	0.8	0.83	Bootstrapping	AUC & DCA	Yes
		489	171 (35)		Prior Bx	0.76	0.81			
Radtke, 2019	No (Ex Val)	160	76 (47.5)	6 (2.0)	Heidelberg	0.77	0.86	N/A	AUC & DCA	Yes
		133	38 (28.6)		UCHL	0.77	0.86			
Tay, 2021	No (Ex Val)	332	96 (28.9)	5 (1.5)	All	0.87	0.89	N/A	AUC & DCA	No
van Leeuwen, 2017	Yes	393	149 (37.9)	6 (1.5)	All	0.819	0.897	Bootstrapping	AUC & DCA	Yes
Zhang, 2020	Yes	573	165 (28.8)	N/I	All	0.82	0.891	Non-random split data	AUC & DCA	Yes

*N/I* no information, *N/A* not applicable, *Clin* Clinical, *DCA* decision curve analysis.

^a^Value from external validation.

**Table 3:** Predictors included in MRI-based risk calculators across studies.

Author, Year	Age	PSA	PI-RADS	PV	DRE	Family hx	Race	Bx history	PSAD	PNB	TRUS	f/t PSA	Ethnicity
Alberts, 2018	✓	✓	✓	✓	✓			✓					
Bhat, 2019	✓	✓	✓		✓	✓	✓	✓					
Falagario, 2021	✓	✓	✓	✓	✓	✓			✓				
Fang, 2016	✓	✓	✓	✓	✓						✓		
Hagens, 2022	✓	✓	✓	✓	✓			✓					
Kim, 2016	✓	✓	✓		✓	✓	✓	✓					
Kinnaird, 2022	✓	✓	✓	✓	✓	✓	✓	✓	✓				✓
Mehralivand, 2018	✓	✓	✓	✓	✓	✓	✓	✓	✓	✓			
Mortezavi, 2020	✓	✓	✓	✓	✓			✓		✓			
Parekh, 2022	✓	✓	✓	✓		✓	✓	✓	✓	✓			
Patel, 2023	✓	✓	✓	✓	✓			✓					
Radtke, 2017	✓	✓	✓	✓	✓								
Radtke, 2019	✓	✓	✓	✓	✓								
Tay, 2021	✓	✓	✓	✓	✓			✓					
van Leeuwen, 2017	✓	✓	✓	✓	✓			✓					
Zhang, 2020	✓	✓	✓		✓				✓			✓	

✓Indicates that the predictor was included in the risk calculator model.

*PSA* prostate-specific antigen, *PI-RADS* Prostate Imaging Reporting and Data System, *PV* prostate volume, *DRE* digital rectal examination, *Family hx* family history, *Bx history* prior biopsy history, *PSAD* PSA density, *PNB* prior negative biopsy, *TRUS* transrectal ultrasound, *f/t PSA* free-to-total PSA ratio.

### 3.4. Quality assessment

**Fig. 2:**
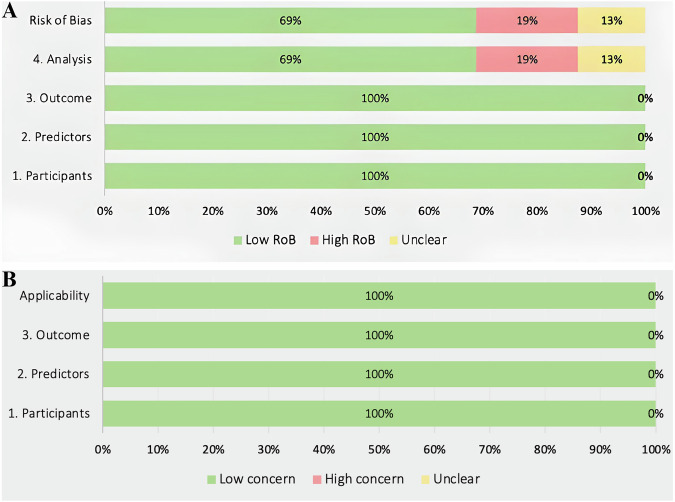
Prediction model Risk Of Bias ASsessment Tool (PROBAST) assessment of included studies. **A** Risk of Bias, **B** Applicability.

Eleven of the 16 included studies (69%) were assessed as having a low risk of bias, Fig. 2, and all studies were applicable. Two studies (13%) were assessed as having an unclear risk of bias in their analysis, whereas three studies (19%) were assessed as having a high risk of bias in the analysis section.

### Meta-analysis

#### Clinically significant prostate cancer

**Fig. 3:**
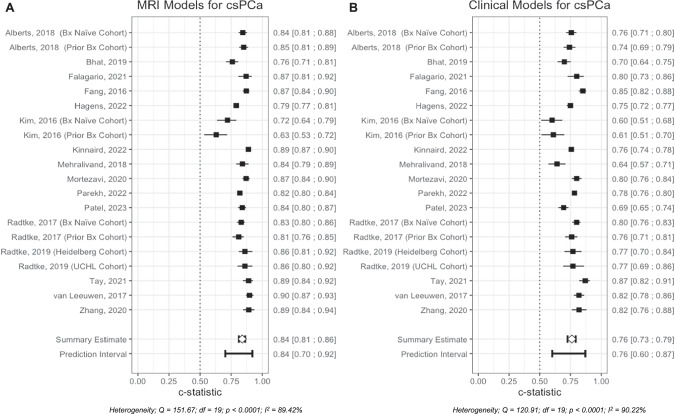
Forest plots of reported AUC for both models for csPCa types. **A** MRI-based RCs for csPCa, **B** Clinical RCs for csPCa.

For the detection of csPCa 20 comparisons were carried out, the pooled AUC estimate for MRI-based RCs (Fig. 3A) showed good discrimination at 0.84 (95% CI: 0.81; 0.86), indicating improved diagnostic accuracy compared to clinical RCs (Fig. 3B), which had a discrimination estimate of 0.76 (95% CI: 0.73; 0.79). Both demonstrated substantial heterogeneity, with *I*² values of 89.42% for MRI-based RCs and 90.22% for clinical RCs, suggesting significant variability in performance across the included studies.

#### All prostate cancer

**Fig. 4:**
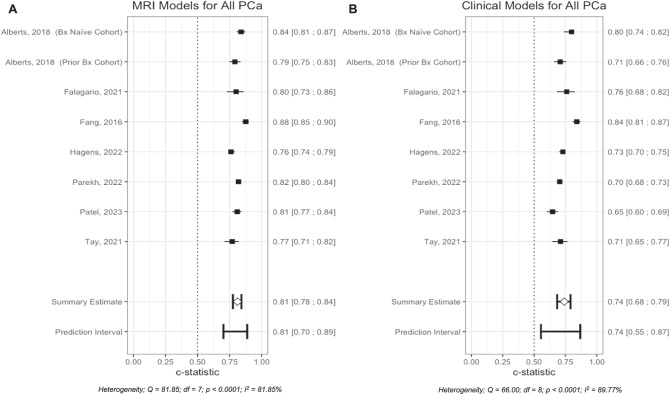
Forest plots of reported AUC for both models and All PCa types. **A** MRI-based RCs for All PCa, **B** Clinical RCs for All PCa.

In the assessment of all PCa eight comparisons were performed, MRI-based RCs (Fig. 4A) outperformed clinical RCs (Fig. 4B), with a pooled AUC estimate of 0.81 (95% CI: 0.78; 0.84), compared to 0.74 (95% CI: 0.68; 0.79). Similar to csPCa findings, both exhibited substantial heterogeneity, with *I*² values of 81.85% for MRI-based RCs and 91.51% for clinical RCs, further indicating variability in diagnostic performance across studies.

#### Difference in discrimination

**Fig. 5:**
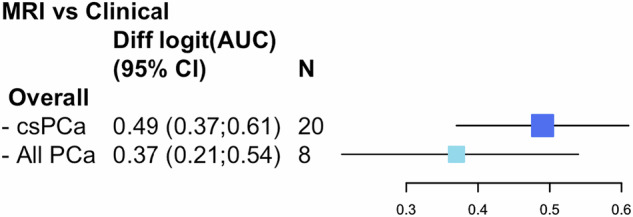
Meta-analysis of the difference in logit(AUC) between MRI and clinical RCs for both types of PCa.

The pooled difference in logit(AUC) between MRI-based and clinical RCs, shown in Fig. 5, was 0.49 units (95% CI: 0.37–0.61), indicating that MRI-based RCs demonstrate an enhanced ability to discriminate PCa cases compared to traditional clinical RCs. Each 5% increase in logit(AUC) corresponds to an approximate 0.01 increase in AUC.

For the detection of all PCa, the pooled difference in logit(AUC) was 0.37 units (95% CI: 0.21–0.54), suggesting that MRI-based RCs also outperform clinical RCs across a broader patient population, although the improvement is larger for csPCa.

#### Subgroup meta-analyses

Subgroup meta-analyses were conducted to investigate sources of heterogeneity for csPCa, focusing on the proportion of PI-RADS 3 cases, sample size, and validation type. Twenty studies were compared across three subgroups. Subgroup results are shown in Supplementary Table 1.

##### Proportion of PI-RADS 3 cases

For studies with a low proportion of PI-RADS 3 cases (median split <20.7%), seven studies for both clinical and MRI models, the pooled AUC for MRI-based RCs was 0.85 (95% CI: 0.80–0.89; *I*² = 81.25%), compared to 0.77 (95% CI: 0.70–0.82; *I*² = 89.97%) for clinical RCs. In studies with a high proportion of PI-RADS 3 cases, MRI-based RCs demonstrated a similar pooled AUC of 0.85 (95% CI: 0.81–0.89; *I*² = 86.50%), while clinical RCs had an AUC of 0.74 (95% CI: 0.67–0.80; *I*² = 89.79%). Suggesting that MRI-based RCs maintain consistent performance regardless of PI-RADS 3 case prevalence.

##### Sample size

When stratified by sample size, smaller studies (median split <428.5 participants) showed a pooled AUC of 0.82 (95% CI: 0.76–0.87; *I*² = 86.85%) for MRI-based RCs and 0.74 (95% CI: 0.67–0.80; *I*² = 87.21%) for clinical RCs. Larger studies (median split ≥ 428.5 participants) reported higher discrimination estimates, with MRI-based RCs achieving a pooled AUC of 0.85 (95% CI: 0.82–0.87; *I*² = 84.00%) and clinical RCs reaching 0.78 (95% CI: 0.74–0.81; *I*² = 86.22%). larger studies showed higher overall discrimination with consistently better performance for MRI-based RCs, suggesting more reliable results in larger cohorts.

##### Validation type

By validation type, internally validated models yielded a pooled AUC of 0.84 (95% CI: 0.80–0.87; *I*² = 90.33%) for MRI-based RCs and 0.77 (95% CI: 0.69–0.84; *I*² = 89.16%) for clinical RCs. Externally validated models showed similar trends, with MRI-based RCs achieving an AUC of 0.85 (95% CI: 0.82–0.89; *I*² = 72.39%) and clinical RCs reaching 0.77 (95% CI: 0.69–0.83; *I*² = 87.00%). Both internal and external validation analyses demonstrated consistently superior discrimination for MRI-based RCs compared to clinical RCs, suggesting robust performance across validation settings.

##### Discrimination advantage of MRI in subgroup meta-analyses

To further assess the impact of MRI in model performance, we compared the differences in discrimination between MRI-based and clinical RCs across each subgroup, shown in Table 4. The pooled differences in logit(AUC) between MRI-based and clinical RCs were statistically significant across all subgroups, consistently favouring MRI-based RCs. The largest difference was seen in studies with a high proportion of PI-RADS 3 cases (0.55 units; 95% CI: 0.20–0.90; *p* = 0.0098; *I*² = 89.8%) and in externally validated models (0.58 units; 95% CI: 0.31–0.86; p = 0.0016; *I*² = 97.1%).

**Table 4:** Subgroup meta-analysis: Differences in logit(AUC) between MRI-based and clinical risk calculators for the detection of csPCa.

Difference in logit(AUC) for csPCa						
Subgroup	Qualifyer	Estimate	95% CI		*P* val	*I*^2^ (%)
PIRADS 3	*Low*	0.5	0.23	0.76	0.0034	98.7
	*High*	0.59	0.41	0.76	0.0002	0
Sample Size	*Small*	0.46	0.3	0.62	<0.0001	75.5
	*Large*	0.52	0.35	0.69	<0.0001	58.9
Validation	*Internal*	0.49	0.36	0.62	<0.0001	64.5
	*External*	0.58	0.31	0.86	0.0016	97.1

Estimates represent the pooled difference in logit(AUC) with corresponding 95% confidence intervals (CI), *p*-values, and heterogeneity (*I*²) for each subgroup. Subgroups include PI-RADS 3 prevalence (low vs. high), sample size (small vs. large), and validation type (internal vs. external).

## Discussion

This review aimed to evaluate the discriminative ability of RCs that integrate MRI findings with clinical risk factors for PCa diagnoses. To achieve this, we synthesised evidence from multiple studies focusing on the development, validation, and performance of MRI-based RCs. Sixteen studies published between 2016 and 2023, representing research from Europe, Asia, Australia, and North America, were included in the review.

This study focused specifically on the discrimination of MRI-based RCs in comparison to a wider focus on all RCs in the review by Denijs et al. [18]. In addition, our meta-analysis quantifies the impact of including MRI for all PCa and clinically significant PCa diagnosis.

### Diagnostic performance and clinical decision-making

The meta-analysis demonstrated that MRI-based RCs had significantly improved discriminative ability compared to clinical RCs, with the largest effect observed for the diagnosis of csPCa. The results, based on data from 20 cohorts for csPCa and eight cohorts for all PCa, indicate that MRI-based RCs consistently outperformed clinical RCs in their ability to differentiate PCa patients according to their outcome. The pooled analysis demonstrated an improvement in the ability to distinguish between csPCa and non-significant cases, with a difference in logit(AUC) of 0.49 units (95% CI: 0.37–0.61), highlighting their enhanced performance in identifying high-risk cases. For all PCa cases, the improvement in discriminative power, though still significant, was slightly lower, with a pooled difference in logit(AUC) of 0.37 units (95% CI: 0.21–0.54). These increases in AUC may be clinically meaningful, particularly for csPCa. However, the true clinical utility of such improvements is best evaluated using decision-analytic methods such as Decision Curve Analysis (DCA), which assesses the net benefit across a range of threshold probabilities [46]. An AUC increase from 0.76 to 0.84 could reduce unnecessary biopsies and enable earlier intervention for aggressive cancers. Even modest improvements (e.g. 0.05) can be clinically meaningful [46], though DCA is required to confirm this. The increased diagnostic accuracy of MRI-based RCs could play an important role in enhancing clinical decision-making and patient management by reducing the occurrence of false positives and negatives. This allows for more accurate treatment decisions and better allocation of medical resources.

Notably, MRI-based RCs appear especially valuable for detecting csPCa, which presents a higher risk of morbidity and necessitates timely interventions. Their use in identifying csPCa may lead to reduced unnecessary interventions and improved patient outcomes.

Moreover, the analysis highlights that each 5% increase in logit(AUC) corresponds to an approximate 0.01 increase in AUC. In the context of PCa diagnostics, where precise identification of clinically significant diseases is paramount.

To explore whether study-level characteristics influenced the observed differences in discrimination, we conducted subgroup meta-analyses based on the proportion of PI-RADS 3 cases, sample size, and model validation type. Across all subgroups, MRI-based RCs maintained superior discrimination over clinical RCs. The largest effect was observed in studies with a higher proportion of PI-RADS 3 cases (difference in logit(AUC): 0.59; 95% CI: 0.41–0.76), suggesting that MRI-based RCs may be particularly beneficial for patient groups with more equivocal MRI findings. Similarly, externally validated models showed a large performance improvement (difference in logit(AUC): 0.58; 95% CI: 0.31–0.86). While larger studies reported higher absolute AUCs for both MRI-based and clinical RCs, the relative discriminative advantage of MRI-based RCs remained consistent across study sizes.

A key barrier to clinical implementation of the RCs is the lack of validated decision thresholds for a prostate biopsy. Most models do not specify clear risk cut-off above which biopsy is recommended or below which it can be safely avoided. This limits their practical utility and hampers their integration into clinical decision-making pathways. Future research should focus on defining and validating such thresholds across diverse populations to ensure safe, consistent, and evidence-based application in practice.

MRI-based RCs do not merely replicate the diagnostic information provided by MRI but enhance it by integrating imaging findings with clinical and demographic variables such as PSA level, DRE results, age, and family history. This combined approach offers a more accurate risk estimation than MRI alone, particularly in equivocal cases such as PI-RADS 3. In contemporary urological practice, this quantification of risk is of practical benefit, supporting clearer communication with patients and improving consistency across clinical settings, especially where MRI interpretation may vary due to radiologist experience or institutional resources.

MRI-based RCs may be particularly valuable in lower-volume centres supporting more equitable and data-driven biopsy decision-making across diverse healthcare settings. A recent study by Nalavenkata et al. [47] reported large international differences in the absolute risk of csPCa for PI-RADS 4 and 5 lesions, ranging from 23 to 68% and 49 to 87%, respectively. In contrast, institutional audits in high-volume centres have reported csPCa rates of approximately 70% for PI-RADS 4 and 90% for PI-RADS 5 lesions. This variability underscores the potential role of MRI-based RCs in standardising risk assessment and guiding biopsy decisions where interpretative consistency may be lower.

### Impact on resource allocation

These findings suggest that MRI-based RCs could improve PCa risk assessment by more accurately identifying high-risk patients, reducing unnecessary biopsies, and optimising resource allocation. Prioritising high-risk patients for biopsy and treatment may also help ease the workload for radiologists, urologists, and pathology services.

The economic burden of PCa is significant, with unnecessary biopsies, overtreatment, and hospital admissions contributing substantially to these costs [48]. MRI-based RCs offer economic benefits by lowering healthcare costs associated with overdiagnosis and invasive procedures. In Ireland, for example, each prostate biopsy costs €432 [48], representing a significant financial burden to the healthcare system. By reducing the need for such procedures, MRI-based RCs can contribute to more cost-effective and sustainable healthcare delivery.

Importantly, MRI-based RCs also offer practical advantages over genetic tests and biomarker-based assays such as the 4Kscore, PHI, and SelectMDx. These tools, while promising, are often costly, may require biological samples with specialised processing, and may not be widely accessible in all clinical settings [49]. In contrast, MRI-based RCs can be implemented more broadly using readily available clinical and imaging data, offering an efficient and scalable solution.

### Clinical utility and future integration

MRI-based RCs, often delivered as web-based calculators or embedded in clinical systems, represent an evolution of traditional nomograms, combining user-friendliness with modern imaging data. Nomograms remain widely used tools for PCa risk prediction, offering intuitive, visual representations of multivariate models. However, as Lombardo and De Nunzio [50] argue, there is a need for greater external validation and harmonisation across different models to support broader clinical uptake.

Despite their benefits, MRI-based RCs have not been widely adopted in routine clinical practice. A key challenge is the lack of seamless integration into clinical workflows. As Anger et al. [51] highlight, embedding RCs into electronic health records, standardising their use, and ensuring real-time availability during consultations are critical to facilitating their uptake. Without such integration, even well-validated tools may be underutilised. Therefore, successful implementation of MRI-based RCs will require attention not only to model performance but also to operational feasibility, user interface design, and integration within clinical information systems.

### Strengths and limitations

The strengths of this study include a comprehensive search across multiple databases (Embase, Medline, Scopus, Cochrane Library, and Web of Science) and the use of PROBAST risk of bias assessment to ensure transparency and quality evaluation. This meta-analysis pooled data from 16 studies, providing a robust synthesis of the diagnostic performance of MRI-based RCs. Sample sizes ranged from 221 to 2363 across diverse regions, increasing generalisability.

Despite these strengths, some limitations should be acknowledged. Most included studies were retrospective, potentially affecting data quality and standardisation. Heterogeneity was high across included studies. This likely reflects variability in demographics, tumour characteristics, study design, and statistical methods [52]. Additionally, heterogeneity could have been introduced by technical factors such as differences in MRI acquisition protocols, interpretation variability between radiologists, and the use of different PI-RADS versions or scoring practices. Given that PCa exhibits a wide spectrum of biological behaviours, understanding this heterogeneity is crucial for the development of accurate diagnostic models. The development of cohort-specific RCs offers a potential strategy for addressing variability. Alternatively, recalibrating existing RCs for particular clinical settings or populations may improve their calibration and clinical utility [53]. The conducted subgroups only had a modest impact on reducing heterogeneity. For example, while heterogeneity was somewhat lower in larger studies (MRI-based RCs *I*²: 84.0%) and externally validated models (*I*²: 77.5%), it remained high in most strata. An exception was observed in the difference-in-discrimination analysis for studies with a high proportion of PI-RADS 3 cases, where heterogeneity was absent (I²: 0%), suggesting that specific study characteristics can explain heterogeneity in some contexts. Nonetheless, residual heterogeneity persisted despite subgrouping, likely reflecting differences in patient populations, PCa prevalence, radiologist expertise and healthcare settings, limiting the certainty of pooled effect estimates and reinforcing the need for more standardised reporting and study designs in future research.

### Further research

In this study, we identified that 20.3% of all PI-RADS scores were classified as category 3. Although there is growing evidence that multivariate RCs can improve risk assessment for patients with PI-RADS 3 lesions [54], managing these cases remains challenging as these cases often present with diagnostic uncertainty.

Prostate-specific membrane antigen positron emission tomography (PSMA PET) imaging has emerged as a potentially valuable adjunct in this setting. By targeting prostate-specific membrane antigen expression, PSMA PET allows more sensitive detection of PCa lesions than conventional imaging. Evidence from the PRIMARY trial [55], suggests that diagnostic PSMA PET may offer a cost-effective and accurate method to triage PI-RADS 1 to 3 lesions, particularly in health systems where PET imaging is accessible.

AI offers promising opportunities to enhance MRI-based RCs by improving image interpretation and risk prediction. Baydoun et al. [56] highlight the use of machine learning models, including support vector machines, random forests, and deep learning techniques like convolutional neural networks (CNNs), for tasks such as automated lesion detection and predictive modelling. These AI-driven tools can help reduce inter-reader variability and support more consistent risk stratification when integrated into RCs. AI-based nomograms, such as those described by Wang et al. [57], are also being developed to predict clinically relevant outcomes like lymph node metastasis. However, challenges remain around generalisability across diverse populations and the limited transparency of some complex AI models, which may hinder clinical implementation. As PCa diagnosis increasingly involves high-dimensional data, AI will likely play an expanding role in risk prediction and clinical decision-making.

Prostate-specific antigen density (PSAD) is increasingly used to stratify PI-RADS 3 cases, with higher values indicating biopsy and lower values supporting surveillance [58, 59]. Furthermore, the Likert scoring system has been proposed as an alternative or complement to PI-RADS [60], with studies showing that it results in fewer equivocal scores, reducing them by approximately 8.4% [61]. The Calculated Adjustment of PI-RADS Equivocal Score (CAPES) tool [62] further improved clarity, reducing equivocal scores by approximately 21.9% compared to PI-RADS [61], potentially offering a more clear diagnostic guideline.

Emerging research on novel biomarkers also may offer promise for advancing the diagnosis and management of PCa [49]. However, the clinical utility of such biomarkers remains uncertain, and rigorous external validation in prospective cohorts is necessary before their integration into MRI-based RCs can be recommended.

## Conclusion

Compared with traditional clinical RCs, MRI-based RCs demonstrate a significantly enhanced ability to discriminate between csPCa and PCa. By incorporating MRI data, these calculators provide more precise stratification of patients based on the likelihood of csPCa. This refined stratification is crucial in identifying patients who may benefit from biopsy or active treatment while safely avoiding unnecessary interventions in low-risk individuals.

The integration of MRI-based RCs into clinical practice has the potential to enhance decision making by offering a personalised approach to PCa management. Patients can receive more tailored counselling with a clearer understanding of their risk profile, thereby improving shared decision making. Ultimately, MRI-based RCs could reduce overtreatment and enhance the detection of aggressive cancers at earlier and more treatable stages, leading to improved patient outcomes and more efficient resource utilisation in healthcare settings.

## Supplementary information

Supplementary File

## Data Availability

Dataset is published on Mendeley Data [63].

## Code availability

Full code is available in the supplementary file and as a downloadable file with the dataset [63].
