# Supplementary material for: Clinical impact of MRI-based risk calculators for prostate cancer diagnosis: a systematic review and meta-analysis
Source: Prostate Cancer Prostatic Dis. 2025 Aug 26;29(2):247–57. doi: 10.1038/s41391-025-01014-2 (PMC13190291; doi:10.1038/s41391-025-01014-2)
Supplement: Supplementary file 1 — Supplementary File [file 41391_2025_1014_MOESM1_ESM.docx]

**Supplementary file**

***Publication Bias***

Figure S1 shows the funnel plot of MRI-based RCs for csPCa The plot is reasonably symmetric (p-value from Egger’s test of 0.57) with no significant evidence of funnel plot asymmetry.. Figure S2 shows the funnel plot of clinical RCs for csPCa, which is also reasonably symmetric (p-value from Egger’s test of 0.89) with no significant evidence of funnel plot asymmetry. Overall, there appeared to be no evidence of publication bias in the assessed studies.


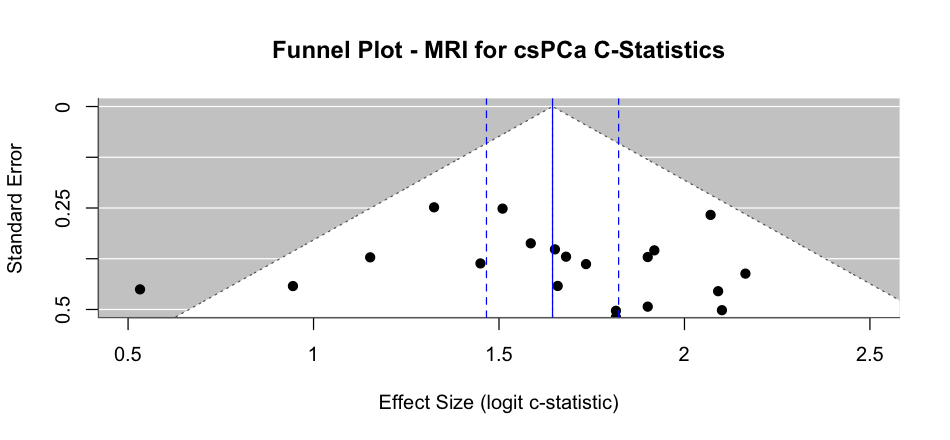


Figure S1. Funnel plot of MRI RCs for csPCa


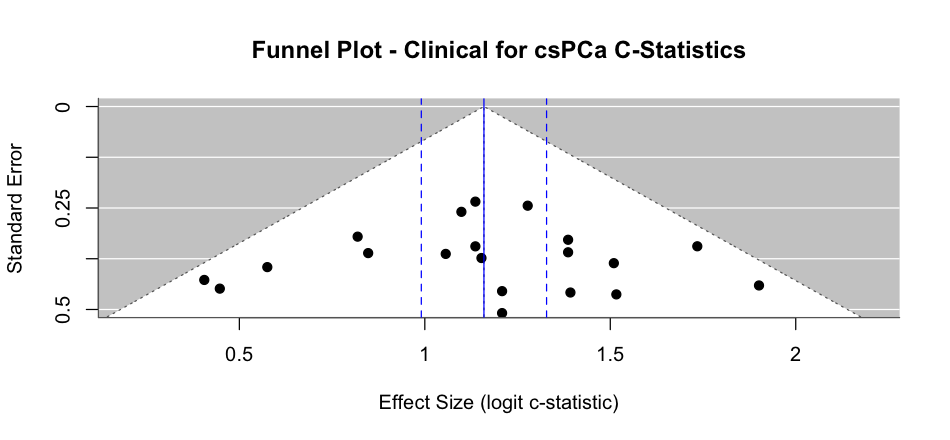


Figure S2. Funnel plot for clinical RCs for csPCa

Table S1. Subgroup meta-analysis results

| **Subgroup Meta-Analyses for csPCa** | | | | | | | | | | |
| --- | --- | --- | --- | --- | --- | --- | --- | --- | --- | --- |
| **Subgroup** | **Qualifier** | **Model** | **No. Studies** | **Estimate** | **95% CI** | | **Prediction Interval** | | **I^2 (%)** |  |
| **PIRADS 3** | ***Low*** | *Clinical* | 7 | 0.78 | 0.70 | 0.84 | 0.54 | 0.91 | 89.97 |  |
|  |  | *MRI* | 7 | 0.85 | 0.80 | 0.89 | 0.70 | 0.93 | 81.25 |  |
|  | ***High*** | *Clinical* | 5 | 0.77 | 0.69 | 0.83 | 0.53 | 0.91 | 92.69 |  |
|  |  | *MRI* | 5 | 0.85 | 0.80 | 0.89 | 0.67 | 0.94 | 89.87 |  |
| **Sample Size** | ***Small*** | *Clinical* | 10 | 0.74 | 0.67 | 0.80 | 0.49 | 0.90 | 87.91 |  |
|  |  | *MRI* | 10 | 0.82 | 0.76 | 0.87 | 0.59 | 0.94 | 86.85 |  |
|  | ***Large*** | *Clinical* | 10 | 0.78 | 0.74 | 0.81 | 0.66 | 0.86 | 86.22 |  |
|  |  | *MRI* | 10 | 0.85 | 0.82 | 0.87 | 0.76 | 0.91 | 84.00 |  |
| **Validation** | ***Internal*** | *Clinical* | 16 | 0.76 | 0.72 | 0.79 | 0.60 | 0.87 | 89.16 |  |
|  |  | *MRI* | 16 | 0.84 | 0.80 | 0.87 | 0.68 | 0.93 | 90.33 |  |
|  | ***External*** | *Clinical* | 7 | 0.77 | 0.69 | 0.84 | 0.53 | 0.91 | 87.00 |  |
|  |  | *MRI* | 7 | 0.85 | 0.82 | 0.89 | 0.73 | 0.93 | 72.39 |  |

No. of Studies: Number of Studies; I^2^ = Heterogeneity

**Search strategy – Medline, Embase**

*Search Query – 482 results*

'prostat* cancer':ti,ab,kw AND ('magnetic resonance imaging':ti,ab,kw OR 'mri':ti,ab,kw OR mpmri:ti,ab,kw OR 'multiparametric magnetic resonance imaging':ti,ab,kw) AND ('auc' OR 'area under the curve') AND diagnos*:ti,ab,kw AND (model*:ti,ab,kw OR 'risk calculator':ti,ab,kw)

**Keywords by fields**

- prostat* cancer in Title
- prostat* cancer in Abstract
- prostat* cancer in Author keyword
- magnetic resonance imaging in Title
- magnetic resonance imaging in Abstract
- magnetic resonance imaging in Author keyword
- mri in Title
- mri in Abstract
- mri in Author keyword
- mpmri in Title
- mpmri in Abstract
- mpmri in Author keyword
- multiparametric magnetic resonance imaging in Title
- multiparametric magnetic resonance imaging in Abstract
- multiparametric magnetic resonance imaging in Author keyword
- auc in All Fields
- area under the curve in All Fields
- diagnos* in Title
- diagnos* in Abstract
- diagnos* in Author keyword
- model* in Title
- model* in Abstract
- model* in Author keyword
- risk calculator in Title
- risk calculator in Abstract
- risk calculator in Author keyword

**Restrictions**

No date range restrictions were applied.

English language restriction applied.

Restrictions on publication type were applied to exclude:

-Pre-prints

-Editorials

-Discussions

-Commentaries

-Grey literature

-Conference abstracts

-Full text not available

R code for analysis

#######################################################################

# R Code to accompany MRI-based risk calculators for prostate #

# cancer diagnosis: A systematic review and meta-analysis #

#######################################################################

### Pooled AUC Meta-Analysis ###

# libraries

library(readxl)

library(metamisc)

library(ggplot2)

library(metafor)

# Read in data

aucmet <- read_excel("Downloads/Supplementary Data Extraction File.xlsx",,

sheet = "Meta-data",

col_types = c("text", rep("numeric", 22), "text"))

View(aucmet)

# Meta-analysis of MRI AUC for csPCa

fit_cs_mri <- valmeta(

cstat = cs_auc_mri,

cstat.se = se,

cstat.cilb = cs_cilb_mri,

cstat.ciub = cs_ciub_mri,

cstat.cilv = 0.95,

N = n,

O = O.ev,

slab = study,

data = aucmet,

)

# Plot the forest plot

p1 <- plot(fit_cs_mri, sort = "theta.slab",

title = 'MRI Models for csPCa',) +

theme(plot.title = element_text(hjust = 0.5)) #Centre title

print(p1)

# Extract heterogeneity statistics from the model

het_mri_cs <- data.frame(

I2 = round(fit_cs_mri$fit$I2,2), # I^2 statistic

Q = round(fit_cs_mri$fit$QE,2), # Cochran's Q-statistic

DF = round(fit_cs_mri$fit$dfs,2), # Degrees of freedom

P_Val = ifelse(fit_cs_mri$fit$QEp < 0.0001, "<0.0001") # P-value for heterogeneity test

)

het_mri_cs #print heterogeneity results

# Publication Bias assessment

# Extract effect sizes (yi) and variances (vi)

yi_cs_mri <- fit_cs_mri$fit$yi # Log-transformed c-statistics (effect sizes)

vi_cs_mri <- fit_cs_mri$fit$vi # Variance for each effect size

range(yi_cs_mri)

# Calculate standard errors (sei) as the square root of variance

sei_cs_mri <- sqrt(vi_cs_mri)

range(sei_cs_mri)

# Calculate 95% CI for the pooled effect

pooled_effect_cs_mri <- as.vector(fit_cs_mri$fit$b) # Pooled effect size

pooled_se_cs_mri <- sqrt(fit_cs_mri$fit$vb) # Pooled standard error

ci_lower_cs_mri <- pooled_effect_cs_mri - 1.96 * pooled_se_cs_mri

ci_upper_cs_mri <- pooled_effect_cs_mri + 1.96 * pooled_se_cs_mri

# Create funnel plot

funnel(yi_cs_mri, sei_cs_mri,

main = "Funnel Plot - MRI for csPCa C-Statistics",

xlab = "Effect Size (logit c-statistic)",

ylab = "Standard Error",

refline = pooled_effect_cs_mri,

xlim = c(0.5, 2.5), # Adjust based on the range of yi_cs_mri

ylim = c(0, 0.5)) # Adjust based on sei_cs_mri range

# Add confidence intervals as vertical lines

abline(v = c(ci_lower_cs_mri, pooled_effect_cs_mri, ci_upper_cs_mri),

lty = c(2, 1, 2), col = "blue")

#Egger's test for funnel plot asymmetry

pb_cs_mri <- fat(yi_cs_mri, vi_cs_mri)

print(pb_cs_mri) # print result

# Meta-analysis of Clinical AUC for csPCa

aucmet[!is.na(aucmet$all_auc_mri),] # Subset

fit_cs_clin <- valmeta(

cstat = cs_auc_clin,

cstat.se = se,

cstat.cilb = cs_cilb_clin,

cstat.ciub = cs_ciub_clin,

cstat.cilv = 0.95,

N = n,

O = O.ev,

slab = study,

data = aucmet,

)

# Plot the forest plot

p2 <- plot(fit_cs_clin, sort = "theta.slab",

title = 'Clinical Models for csPCa',) +

theme(plot.title = element_text(hjust = 0.5))

print(p2)

# Extract heterogeneity statistics from the model and store as data frame

het_clin_cs <- data.frame(

I2 = round(fit_cs_clin$fit$I2,2), # I^2 statistic

Q = round(fit_cs_clin$fit$QE,2), # Cochran's Q-statistic

DF = round(fit_cs_clin$fit$dfs,2), # Degrees of freedom

P_Val = ifelse(fit_cs_clin$fit$QEp < 0.0001, "<0.0001") # P-value for heterogeneity test

)

het_clin_cs #print heterogeneity results

# Publication Bias assessment

# Extract effect sizes (yi) and variances (vi)

yi_cs_clin <- fit_cs_clin$fit$yi # Log-transformed c-statistics (effect sizes)

vi_cs_clin <- fit_cs_clin$fit$vi # Variance for each effect size

range(yi_cs_clin)

# Calculate standard errors (sei) as the square root of variance

sei_cs_clin <- sqrt(vi_cs_clin)

range(sei_cs_clin)

# Calculate 95% CI for the pooled effect

pooled_effect_cs_clin <- as.vector(fit_cs_clin$fit$b) # Pooled effect size

pooled_se_cs_clin <- sqrt(fit_cs_clin$fit$vb) # Pooled standard error

ci_lower_cs_clin <- pooled_effect_cs_clin - 1.96 * pooled_se_cs_clin

ci_upper_cs_cin <- pooled_effect_cs_clin + 1.96 * pooled_se_cs_clin

# Create funnel plot

funnel(yi_cs_clin, sei_cs_clin,

main = "Funnel Plot - Clinical for csPCa C-Statistics",

xlab = "Effect Size (logit c-statistic)",

ylab = "Standard Error",

refline = pooled_effect_cs_clin,

xlim = c(0.2, 2.2), # Adjust to the range of yi_cs_mri

ylim = c(0, 0.5)) # Adjust based on sei_cs_mri range

# Add confidence intervals as vertical lines

abline(v = c(ci_lower_cs_clin, pooled_effect_cs_clin, ci_upper_cs_cin),

lty = c(2, 1, 2), col = "blue")

#Egger's test for funnel plot asymmetry

pb_cs_clin <- fat(yi_cs_clin, vi_cs_clin)

print(pb_cs_clin) # print result

# Meta-analysis of MRI AUC for all PCa

fit_all_mri <- valmeta(

cstat = all_auc_mri,

cstat.se = se,

cstat.cilb = all_cilb_mri,

cstat.ciub = all_ciub_mri,

cstat.cilv = 0.95,

N = n,

O = O.ev,

slab = study,

data = aucmet[!is.na(aucmet$all_auc_mri),],

)

# Plot the forest plot

p3 <- plot(fit_all_mri, sort = "theta.slab",

title = 'MRI Models for All PCa',) +

theme(plot.title = element_text(hjust = 0.5))

print(p3)

# Extract heterogeneity statistics from the model

het_mri_all <- data.frame(

I2 = round(fit_all_mri$fit$I2,2), # I^2 statistic

Q = round(fit_all_mri$fit$QE,2), # Cochran's Q-statistic

DF = round(fit_all_mri$fit$dfs,2), # Degrees of freedom

P_Val = ifelse(fit_all_mri$fit$QEp < 0.0001, "<0.0001") # P-value for heterogeneity test

)

het_mri_all #print heterogeneity results

# Meta-analysis of Clinical AUC for all PCa

fit_all_clin <- valmeta(

cstat = all_auc_clin,

cstat.se = se,

cstat.cilb = all_cilb_clin,

cstat.ciub = all_ciub_clin,

cstat.cilv = 0.95,

N = n,

O = O.ev,

slab = study,

data = aucmet[!is.na(aucmet$all_auc_clin),],

)

# Plot the forest plot

p4 <- plot(fit_all_clin, sort = "theta.slab",

title = 'Clinical Models for All PCa',) +

theme(plot.title = element_text(hjust = 0.5))

print(p4)

# Extract heterogeneity statistics from the model

het_clin_all <- data.frame(

I2 = round(fit_all_clin$fit$I2,2), # I^2 statistic

Q = round(fit_all_clin$fit$QE,2), # Cochran's Q-statistic

DF = round(fit_all_clin$fit$dfs,2), # Degrees of freedom

P_Val = ifelse(fit_all_clin$fit$QEp < 0.0001, "<0.0001") # P-value for heterogeneity test

)

print(het_clin_all) #print heterogeneity results

## Difference in AUC Meta-analysis ##

# Libraries

library(boot) # logit function

library(nlme)

library(rmeta) # forest plot

library(insight)

## Calculate logit difference

# csPCa

AUC.difflogit_cs <- logit(aucmet$cs_auc_mri) - logit(aucmet$cs_auc_clin)

# All PCa

AUC.difflogit_all <- logit(aucmet$all_auc_mri[!is.na(aucmet$all_auc_mri)]) -

logit(aucmet$all_auc_clin[!is.na(aucmet$all_auc_mri)])

#Set names of studies

Paper_cs <- aucmet$study

Paper_all <- aucmet$study[!is.na(aucmet$all_auc_mri)]

# use sqrt of validation size as weight in the analysis [Pennells et al 2014]

weight_cs = (aucmet$O.ev / sum(aucmet$O.ev))*100

weight_all = (aucmet$O.ev[!is.na(aucmet$all_auc_mri)]/sum(aucmet$O.ev))*100

# Overall analysis for csPCa

fit.cs <- lme(fixed = AUC.difflogit_cs ~ 1, random = ~ 1|Paper_cs,

weights=varFixed(~weight_cs),data=aucmet)

summary(fit.cs)

# Extract estimate and confidence intervals from model

conf_intervals_cs <- intervals(fit.cs, which = "fixed")$fixed

result_cs <- round(conf_intervals_cs, 2)

# Overall analysis for All PCa

fit.all <- lme(fixed = AUC.difflogit_all ~ 1, random = ~ 1|Paper_all,

weights=varFixed(~weight_all),data=aucmet[!is.na(aucmet$all_auc_mri),])

summary(fit.all)

# Extract estimate and confidence intervals from model

conf_intervals_all <- intervals(fit.all, which = "fixed")$fixed

result_all <- round(conf_intervals_all, 2)

# Table of extracted results

results_table <- data.frame(

Model = c("csPCa (Overall)", "All PCa (Overall)"),

Estimate = c(result_cs[1, "est."], result_all[1, "est."]),

Lower_CI = c(result_cs[1, "lower"], result_all[1, "lower"]),

Upper_CI = c(result_cs[1, "upper"], result_all[1, "upper"])

)

# Print the table

print(results_table)

# forest plot with results

forest1table <- cbind(c("MRI vs Clinical",NA,NA," Overall","- csPCa","- All PCa"),

c(NA,"Diff logit(AUC)","(95% CI)",NA,"0.49 (0.37;0.61)",

"0.37 (0.21;0.54)"),

c(NA,NA,"N",NA,20,8))

m <- c(NA,NA,NA,NA,0.49,0.37)

l <- c(NA,NA,NA,NA,0.37,0.21)

u <- c(NA,NA,NA,NA,0.61,0.54)

a <- c("l","l","l")

forestplot(

forest1table,

mean = m,

lower = l,

upper = u,

align = a,

is.summary = c(TRUE, TRUE, TRUE, TRUE, FALSE, FALSE),

clip = c(0.2, 0.7),

zero = 0,

xlog = FALSE,

col = meta.colors(text = "white", axes = "black", box = "skyblue",

line = "black", summary = "royalblue",

zero = "transparent"),

xticks = NULL

)

par(new=TRUE)

m2 <- c(NA,NA,NA,NA,0.49,NA)

forestplot(forest1table,

mean = m2,

lower = l,

upper = u,

align = a,

is.summary = c(TRUE, TRUE, TRUE, TRUE, FALSE, FALSE),

clip = c(0.2,0.7),

xlog = FALSE,

col=meta.colors(box="royalblue",line="black", summary="royalblue",

zero = "transparent"),

xticks= NULL)

par(new=FALSE)

# Publication Bias assessment

# Extract effect sizes (yi) and variances (vi)

yi_cs <- AUC.difflogit_cs # Log-transformed c-statistics (effect sizes)

vi_cs <- 1 / weight_cs # Variance for each effect size

range(yi_cs)

# Calculate standard errors (sei) as the square root of variance

sei_cs <- sqrt(vi_cs)

range(sei_cs)

# Calculate 95% CI for the pooled effect

pooled_effect_cs <- result_cs[1, "est."] # Pooled effect size

lower_ci_cs <- result_cs[1, "lower"]

upper_ci_cs <- result_cs[1, "upper"]

# Create funnel plot

funnel(yi_cs, sei_cs,

main = "Funnel Plot - csPCa AUC Differences",

xlab = "Effect Size (logit c-statistic)",

ylab = "Standard Error",

refline = pooled_effect_cs,

xlim = c(0, 1.2),

ylim = c(0, 1.2))

# Add confidence intervals as vertical lines

abline(v = c(lower_ci_cs, pooled_effect_cs, upper_ci_cs), lty = c(2, 1, 2),

col = "blue")

##Egger's test for funnel plot asymmetry for csPCa

pb_diff_cs <- fat(yi_cs, sei_cs)

print(pb_diff_cs) # print result

### Subgroup Meta-Analysis ###

# libraries

library(readxl)

library(metamisc)

library(ggplot2)

library(metafor)

library(metafor)

library(boot)

# Read in data

aucmet <- read_excel("Downloads/Supplementary Data Extraction File.xlsx",,

sheet = "Meta-data",

col_types = c("text", rep("numeric", 22), "text"))

#View(aucmet)

valdat <- read_excel("Downloads/Supplementary Data Extraction File.xlsx",,

sheet = "Val-data",

col_types = c("text", rep("numeric", 22), "text"))

#View(valdat)

# Validation as factor

valdat$validation <- as.factor(valdat$validation)

# PI-RADS 3 proportion: split at median

aucmet$pirads_group <- ifelse(aucmet$pirads3 <= median(aucmet$pirads3, na.rm = TRUE), "low", "high")

# Sample size: split at median

aucmet$n_group <- ifelse(aucmet$n <= median(aucmet$n, na.rm = TRUE), "small", "large")

# Validation group

validation_group <- table(valdat$validation)

# Subgroups

# For pirads_group = "Low" [subgroup pi_low]

low_pirads<- subset(aucmet,

pirads_group == "low" &

!is.na(cs_auc_mri) &

!is.na(cs_auc_clin))

clin_fit_cs_pi_low <- valmeta(

cstat = cs_auc_clin,

cstat.se = se,

cstat.cilb = cs_cilb_clin,

cstat.ciub = cs_ciub_clin,

N = n,

O = O.ev,

slab = study,

data = low_pirads[!is.na(low_pirads$cs_auc_clin),]

)

mri_fit_cs_pi_low <- valmeta(

cstat = cs_auc_mri,

cstat.se = se,

cstat.cilb = cs_cilb_mri,

cstat.ciub = cs_ciub_mri,

N = n,

O = O.ev,

slab = study,

data = low_pirads[!is.na(low_pirads$cs_auc_mri),]

)

pi_low_c <- clin_fit_cs_pi_low

pi_low_m <- mri_fit_cs_pi_low

plot(pi_low_c, sort = "theta.slab",

title = 'Clinical Models for csPCa PIRADS 3 Prop Low',) +

theme(plot.title = element_text(hjust = 0.5))

plot(pi_low_m, sort = "theta.slab",

title = 'MRI Models for csPCa PIRADS 3 Prop Low',) +

theme(plot.title = element_text(hjust = 0.5))

# For pirads_group = "High" [subgroup pi_high]

high_pirads <- subset(aucmet, pirads_group == "high" &

!is.na(cs_auc_mri) &

!is.na(cs_auc_clin)&

study != "Radtke, 2019 (UCHL Cohort)")

clin_fit_cs_pi_high <- valmeta(

cstat = cs_auc_clin,

cstat.se = se,

cstat.cilb = cs_cilb_clin,

cstat.ciub = cs_ciub_clin,

N = n,

O = O.ev,

slab = study,

data = high_pirads[!is.na(high_pirads$cs_auc_clin),]

)

mri_fit_cs_pi_high <- valmeta(

cstat = cs_auc_mri,

cstat.se = se,

cstat.cilb = cs_cilb_mri,

cstat.ciub = cs_ciub_mri,

N = n,

O = O.ev,

slab = study,

data = high_pirads[!is.na(high_pirads$cs_auc_mri),]

)

pi_high_c <- clin_fit_cs_pi_high

pi_high_m <- mri_fit_cs_pi_high

plot(pi_high_c, sort = "theta.slab",

title = 'Clinical Models for csPCa PIRADS 3 Prop High',) +

theme(plot.title = element_text(hjust = 0.5))

plot(pi_high_m, sort = "theta.slab",

title = 'MRI Models for csPCa PIRADS 3 Prop High',) +

theme(plot.title = element_text(hjust = 0.5))

# Define PIRADS 3 prop models

models <- list(

"Low - Clinical csPCa" = pi_low_c,

"Low - MRI csPCa" = pi_low_m,

"High - Clinical csPCa" = pi_high_c,

"High - MRI csPCa" = pi_high_m

)

# Extract heterogeneity results

het_results_pi <- do.call(rbind, lapply(names(models), function(name) {

fit <- models[[name]]$fit

data.frame(

Subgroup_Model = name,

I2 = round(fit$I2, 2),

Q = round(fit$QE, 2),

DF = round(fit$dfs, 2),

P_Val = ifelse(fit$QEp < 0.0001, "<0.0001", round(fit$QEp, 4)),

row.names = NULL

)

}))

# View results

print(het_results_pi)

########

# For n_group = "small" [subgroup n_small]

small_n <- subset(aucmet, n_group == "small" &

!is.na(cs_auc_mri) &

!is.na(cs_auc_clin))

clin_fit_cs_n_small <- valmeta(

cstat = cs_auc_clin,

cstat.se = se,

cstat.cilb = cs_cilb_clin,

cstat.ciub = cs_ciub_clin,

N = n,

O = O.ev,

slab = study,

data = small_n[!is.na(small_n$cs_auc_clin),]

)

mri_fit_cs_n_small <- valmeta(

cstat = cs_auc_mri,

cstat.se = se,

cstat.cilb = cs_cilb_mri,

cstat.ciub = cs_ciub_mri,

N = n,

O = O.ev,

slab = study,

data = small_n[!is.na(small_n$cs_auc_mri),]

)

n_small_c <- clin_fit_cs_n_small

n_small_m <- mri_fit_cs_n_small

plot(n_small_c, sort = "theta.slab",

title = 'Clinical Models for csPCa Small Sample Size',) +

theme(plot.title = element_text(hjust = 0.5))

plot(n_small_m, sort = "theta.slab",

title = 'MRI Models for csPCa Small Sample Size',) +

theme(plot.title = element_text(hjust = 0.5))

# For n_group = "large" [subgroup n_large]

large_n <- subset(aucmet, n_group == "large"&

!is.na(cs_auc_mri) &

!is.na(cs_auc_clin))

clin_fit_cs_n_large <- valmeta(

cstat = cs_auc_clin,

cstat.se = se,

cstat.cilb = cs_cilb_clin,

cstat.ciub = cs_ciub_clin,

N = n,

O = O.ev,

slab = study,

data = large_n[!is.na(large_n$cs_auc_clin),]

)

mri_fit_cs_n_large <- valmeta(

cstat = cs_auc_mri,

cstat.se = se,

cstat.cilb = cs_cilb_mri,

cstat.ciub = cs_ciub_mri,

N = n,

O = O.ev,

slab = study,

data = large_n[!is.na(large_n$cs_auc_mri),]

)

n_large_c <- clin_fit_cs_n_large

n_large_m <- mri_fit_cs_n_large

plot(n_large_c, sort = "theta.slab",

title = 'Clinical Models for csPCa Large Sample Size',) +

theme(plot.title = element_text(hjust = 0.5))

plot(n_large_m, sort = "theta.slab",

title = 'MRI Models for csPCa Large Sample Size',) +

theme(plot.title = element_text(hjust = 0.5))

# Define Sample Size models

models <- list(

"Small - Clinical csPCa" = n_small_c,

"Small - MRI csPCa" = n_small_m,

"Large - Clinical csPCa" = n_large_c,

"Large - MRI csPCa" = n_large_m

)

# Extract heterogeneity results

het_results_n <- do.call(rbind, lapply(names(models), function(name) {

fit <- models[[name]]$fit

data.frame(

Subgroup_Model = name,

I2 = round(fit$I2, 2),

Q = round(fit$QE, 2),

DF = round(fit$dfs, 2),

P_Val = ifelse(fit$QEp < 0.0001, "<0.0001", round(fit$QEp, 4)),

row.names = NULL

)

}))

# View results

print(het_results_n)

#####

# For validation_group = "Internal" [subgroup val_int]

int_val <- subset(valdat, validation == "Internal"&

!is.na(cs_auc_mri) &

!is.na(cs_auc_clin))

clin_fit_cs_val_int <- valmeta(

cstat = cs_auc_clin,

cstat.se = se,

cstat.cilb = cs_cilb_clin,

cstat.ciub = cs_ciub_clin,

N = n,

O = O.ev,

slab = study,

data = int_val[!is.na(int_val$cs_auc_clin),]

)

mri_fit_cs_val_int <- valmeta(

cstat = cs_auc_mri,

cstat.se = se,

cstat.cilb = cs_cilb_mri,

cstat.ciub = cs_ciub_mri,

N = n,

O = O.ev,

slab = study,

data = int_val[!is.na(int_val$cs_auc_mri),]

)

val_int_c <- clin_fit_cs_val_int

val_int_m <- mri_fit_cs_val_int

plot(val_int_c, sort = "theta.slab",

title = 'Clinical Models for csPCa Internal Validation',) +

theme(plot.title = element_text(hjust = 0.5))

plot(val_int_m, sort = "theta.slab",

title = 'MRI Models for csPCa Internal Validation',) +

theme(plot.title = element_text(hjust = 0.5))

# For validation_group = "External" [subgroup val_ext]

ext_val <- subset(valdat, validation == "External"&

!is.na(cs_auc_mri) &

!is.na(cs_auc_clin))

clin_fit_cs_val_ext <- valmeta(

cstat = cs_auc_clin,

cstat.se = se,

cstat.cilb = cs_cilb_clin,

cstat.ciub = cs_ciub_clin,

N = n,

O = O.ev,

slab = study,

data = ext_val[!is.na(ext_val$cs_auc_clin),]

)

mri_fit_cs_val_ext <- valmeta(

cstat = cs_auc_mri,

cstat.se = se,

cstat.cilb = cs_cilb_mri,

cstat.ciub = cs_ciub_mri,

N = n,

O = O.ev,

slab = study,

data = ext_val[!is.na(ext_val$cs_auc_mri),]

)

val_ext_c <- clin_fit_cs_val_ext

val_ext_m <- mri_fit_cs_val_ext

plot(val_ext_c, sort = "theta.slab",

title = 'Clinical Models for csPCa External Validation',) +

theme(plot.title = element_text(hjust = 0.5))

plot(val_ext_m, sort = "theta.slab",

title = 'MRI Models for csPCa External Validation',) +

theme(plot.title = element_text(hjust = 0.5))

# Define Validation models

models <- list(

"Internal - Clinical csPCa" = val_int_c,

"Internal - MRI csPCa" = val_int_m,

"External - Clinical csPCa" = val_ext_c,

"External - MRI csPCa" = val_ext_m

)

# Extract heterogeneity results

het_results_val <- do.call(rbind, lapply(names(models), function(name) {

fit <- models[[name]]$fit

data.frame(

Subgroup_Model = name,

I2 = round(fit$I2, 2),

Q = round(fit$QE, 2),

DF = round(fit$dfs, 2),

P_Val = ifelse(fit$QEp < 0.0001, "<0.0001", round(fit$QEp, 4)),

row.names = NULL

)

}))

# View results

print(het_results_val)

####

library(nlme)

run_logit_diff_model <- function(data, auc_mri_col, auc_clin_col, outcome_label, subgroup_label) {

data <- data[!is.na(data[[auc_mri_col]]) & !is.na(data[[auc_clin_col]]), ]

if (nrow(data) < 2) {

return(data.frame(

Subgroup = subgroup_label,

Outcome = outcome_label,

Estimate = NA,

Lower_CI = NA,

Upper_CI = NA,

P_value = NA,

I2 = NA

))

}

data$logit_diff <- logit(data[[auc_mri_col]]) - logit(data[[auc_clin_col]])

# Subgroup-specific weights based on relative observed events

data$weights <- (data$O.ev / sum(data$O.ev)) * 100

model <- lme(

fixed = logit_diff ~ 1,

random = ~1 | study,

weights = varFixed(~weights),

data = data

)

# Extract confidence intervals and estimate

ci <- intervals(model, which = "fixed")$fixed

est <- ci[1, "est."]

lower <- ci[1, "lower"]

upper <- ci[1, "upper"]

# Extract p-value

p_val <- summary(model)$tTable[1, "p-value"]

format_pval <- function(p) {

ifelse(p < 0.0001, "<0.0001", sprintf("%.4f", p))

}

# Extract variance components and compute I²

var_comp <- VarCorr(model)

tau2 <- as.numeric(var_comp[1, "Variance"])

sigma2 <- as.numeric(var_comp[2, "Variance"])

I2 <- round(100 * tau2 / (tau2 + sigma2), 1)

data.frame(

Subgroup = subgroup_label,

Outcome = outcome_label,

Estimate = round(est, 2),

Lower_CI = round(lower, 2),

Upper_CI = round(upper, 2),

P_value = format_pval(p_val),

I2 = I2

)

}

# By PI-RADS proportion

results_pirads <- rbind(

run_logit_diff_model(subset(aucmet, pirads_group == "low"),

"cs_auc_mri", "cs_auc_clin", "csPCa", "low"),

run_logit_diff_model(subset(aucmet, pirads_group == "high" & study != "Radtke, 2019 (UCHL Cohort)"),

"cs_auc_mri", "cs_auc_clin", "csPCa", "high")

)

# By sample size group

results_n <- rbind(

run_logit_diff_model(subset(aucmet, n_group == "small"),

"cs_auc_mri", "cs_auc_clin", "csPCa", "small"),

run_logit_diff_model(subset(aucmet, n_group == "large"),

"cs_auc_mri", "cs_auc_clin", "csPCa", "large")

)

# By validation type

results_val <- rbind(

run_logit_diff_model(subset(valdat, validation == "Internal"),

"cs_auc_mri", "cs_auc_clin", "csPCa", "Internal"),

run_logit_diff_model(subset(valdat, validation == "External"),

"cs_auc_mri", "cs_auc_clin", "csPCa", "External")

)

# Print Results

print(results_pirads)

print(results_n)

print(results_val)

# Numeric vectors for forestplot means and confidence intervals.

mean_vals <- c(NA, NA, results_pirads$Estimate)

lower_vals <- c(NA, NA, results_pirads$Lower_CI)

upper_vals <- c(NA, NA, results_pirads$Upper_CI)

# Define which rows are summary rows - here none, so all FALSE except headers.

is_summary <- c(TRUE, FALSE, rep(FALSE, nrow(results_pirads)))

# Alignment for each column

align_cols <- c("l", "l", "r", "r")

# Plot forestplot

forestplot(

labeltext = table_text,

mean = mean_vals,

lower = lower_vals,

upper = upper_vals,

is.summary = is_summary,

align = align_cols,

zero = 0,

xlab = "Difference in logit(AUC)",

col = meta.colors(box = "skyblue", line = "black", summary = "royalblue"),

clip = c(min(lower_vals, na.rm = TRUE), max(upper_vals, na.rm = TRUE)),

xticks = pretty(c(min(lower_vals, na.rm = TRUE), max(upper_vals, na.rm = TRUE)), n = 5)

)

plot_subgroup_forest <- function(results_df, title = "Subgroup Meta-analysis") {

table_text <- rbind(

c("Subgroup", "Outcome", "Diff logit(AUC)", "95% CI"),

c(NA, NA, NA, NA),

cbind(

results_df$Subgroup,

results_df$Outcome,

sprintf("%.2f", results_df$Estimate),

sprintf("(%.2f; %.2f)", results_df$Lower_CI, results_df$Upper_CI)

)

)

mean_vals <- c(NA, NA, results_df$Estimate)

lower_vals <- c(NA, NA, results_df$Lower_CI)

upper_vals <- c(NA, NA, results_df$Upper_CI)

is_summary <- c(TRUE, FALSE, rep(FALSE, nrow(results_df)))

align_cols <- c("l", "l", "r", "r")

forestplot(

labeltext = table_text,

mean = mean_vals,

lower = lower_vals,

upper = upper_vals,

is.summary = is_summary,

align = align_cols,

zero = 0,

xlab = "Difference in logit(AUC)",

main = title,

col = meta.colors(box = "skyblue", line = "black", summary = "royalblue"),

clip = c(min(lower_vals, na.rm = TRUE), max(upper_vals, na.rm = TRUE)),

xticks = pretty(c(min(lower_vals, na.rm = TRUE), max(upper_vals, na.rm = TRUE)), n = 5)

)

}

plot_subgroup_forest(results_pirads, "PI-RADS Subgroup Meta-analysis")

plot_subgroup_forest(results_n, "Sample Size Subgroup Meta-analysis")

plot_subgroup_forest(results_val, "Validation Type Subgroup Meta-analysis")
